# Nonresponse to Interferon-α Based Treatment for Chronic Hepatitis C Infection Is Associated with Increased Hazard of Cirrhosis

**DOI:** 10.1371/journal.pone.0061568

**Published:** 2013-04-25

**Authors:** Myrna L. Cozen, James C. Ryan, Hui Shen, Robert Lerrigo, Russell M. Yee, Edward Sheen, Richard Wu, Alexander Monto

**Affiliations:** 1 Department of Medicine, Veterans Affairs Medical Center and University of California San Francisco, San Francisco, California, United States of America; 2 Department of Epidemiology, Mailman School of Public Health, Columbia University, New York, New York, United States of America; 3 Department of Medicine, Stanford University School of Medicine, Stanford, California, United States of America; Duke University, United States of America

## Abstract

**Background:**

The long-term consequences of unsuccessful interferon-α based hepatitis C treatment on liver disease progression and survival have not been fully explored.

**Methods and Findings:**

We performed retrospective analyses to assess long-term clinical outcomes among treated and untreated patients with hepatitis C virus in two independent cohorts from a United States Veterans Affairs Medical Center and a University Teaching Hospital. Eligible patients underwent liver biopsy during consideration for interferon-α based treatment between 1992 and 2007. They were assessed for the probability of developing cirrhosis and of dying during follow-up using Cox proportional hazards models, stratified by pretreatment liver fibrosis stage and adjusted for known risk factors for cirrhosis and characteristics affecting treatment selection. The major predictor was a time-dependent covariate for treatment outcome among four patient groups: 1) patients with sustained virological response to treatment; 2) treatment relapsers; 3) treatment nonresponders; and 4) never treated patients. Treatment nonresponders in both cohorts had a statistically significantly increased hazard of cirrhosis compared to never treated patients, as stratified by pretreatment liver fibrosis stage and adjusted for clinical and psychosocial risk factors that disproportionately affect patients who were ineligible for treatment (Veterans Affairs HR = 2.35, CI 1.18–4.69, mean follow-up 10 years, and University Hospital HR = 5.90, CI 1.50–23.24, mean follow-up 7.7 years). Despite their increased risk for liver disease progression, the overall survival of nonresponders in both cohorts was not significantly different from that of never treated patients.

**Conclusion:**

These unexpected findings suggest that patients who receive interferon-α based therapies but fail to clear the hepatitis C virus may have an increased hazard of cirrhosis compared to untreated patients.

## Introduction

More than 3.2 million people in the United States (1% of the population) are chronically infected with hepatitis C virus (HCV) [1], [2]. Until 2011, standard antiviral treatment consisted of subcutaneous pegylated interferon-α (IFNα and oral ribavirin (RBV), which failed to achieve a sustained virological response (SVR, or cure) in approximately half of patients. The proportion of treatment failures is greater among patients with HCV genotype 1, the most prevalent HCV subspecies in the U.S. [3], [4], [5], [6]. The recent addition of an oral protease inhibitor, either boceprevir or telaprevir, to pegylated IFNα/RBV treatment for genotype 1 patients, has increased SVR rates to nearly 75% in treatment naïve patients [7], [8], [9]. SVR has been repeatedly associated with reduced rates of cirrhosis, hepatic decompensation and hepatocellular carcinoma, but the long-term impact of treatment failure on liver disease progression has not been fully explored [10], [11], [12].

Among treatment failures, it has been postulated that transient reductions in viral load during treatment or anti-fibrotic effects of IFNα may attenuate liver disease progression [13]. Alternatively, immunostimulatory influences of IFNα could accelerate liver injury in some patients by triggering hepatic inflammation and scarring [14]. Early observational studies suggested altered short-term progression of liver fibrosis in some treated patients who fail to clear HCV [12], [15], [16], [17], [18]. Pockros, et al, pooled data from eight IFNα-based clinical trials that analyzed paired liver biopsy specimens taken immediately prior to treatment and up to 24 weeks post-treatment, but long-term outcomes were not examined [15]. Short term histologic improvement was seen in some, but not all, treatment failures, and fibrosis progressed in some patients [5], [12], [15], [16], [17], [18]. The evidence from these treatment and from recent retreatment trials, such as HALT-C and EPIC^3^, suggests that failed IFNα-based therapy might have either beneficial, null, or detrimental effects on liver related outcomes in HCV treatment failures [19], [20]. There have been no prospective studies, however, comparing long-term clinical outcomes among chronic HCV patients with IFNα-based treatment failure to that of never treated patients. In the present study, we compared long-term clinical outcomes in two independent cohorts of treated and untreated patients with HCV. Our primary aims were to assess the long-term hazards of cirrhosis and death among the following treatment groups: those who achieved SVR, relapsers, nonresponders, and those who were never treated.

## Methods

### Ethics Statement

This study was conducted in accordance with the ethical principles stated in the Declaration of Helsinki and is consistent with good clinical practice and applicable regulatory requirements [21]. Specific approval was granted by the University of California, San Francisco (UCSF) Institutional Review Board and the SFVA Research and Development Committee for this retrospective records review.

### Study Design and Patient Recruitment

We conducted a medical records review of patients with chronic HCV who were first seen at the San Francisco Veterans Affairs (SFVA) Medical Center Liver Clinic between January, 1992 and July 2007. Most patients had been prospectively consented at the time of liver biopsy for inclusion in a longitudinal database. Eligible patients were ≥18 years of age, had documented chronic HCV, underwent a pre-treatment liver biopsy, received follow-up care at the SFVA Liver Clinic for at least one year after the initial visit, and had at least one follow-up liver imaging study, biopsy or clinic visit. Patients were excluded if they were co-infected with either HIV-1 or hepatitis B virus or if they had decompensated cirrhosis, hepatocellular carcinoma, or liver transplantation prior to their first clinic visit. The study was replicated using an independent cohort of HCV patients from the UCSF Liver Clinic to which the same selection criteria were applied. Each cohort is comprised of all patients meeting study eligibility criteria who were evaluated and followed in these two clinics during this time period.

### Assessment of Clinical Parameters

The SFVA and UCSF electronic medical records were the major sources of data for the study. Deaths were confirmed by cross reference with the national Social Security Death Index (SSDI). Data abstraction was performed by two teams, each including one clinician and one research staff member, using a standardized search algorithm. Two additional raters performed data validation on a random sample of patient charts to confirm the reliability of values for select variables.

Clinical parameters including body mass index (BMI), alanine aminotransferase (ALT), and HCV viral load were collected from the electronic medical record on the date closest to the initial liver clinic visit. Follow-up ALT was obtained from the laboratory test taken closest to 52 weeks following the end of treatment for treated patients. To obtain a comparable ALT value for the never treated patients, we used the ALT closest to 170.7 weeks following initial liver clinic visit. This interval corresponds to the average time between the first liver clinic visit and the completion of one year of post therapy follow-up among treated patients.

Psychosocial, demographic, and behavioral risk factors were assessed and recorded by clinic staff at the time of first liver clinic visit. This assessment and subsequent progress notes were used to determine the presence of risk factors affecting treatment eligibility such as current injection drug use, other substance abuse, history of depression and post-traumatic stress disorder (PTSD). Heavy alcohol use was defined as five or more years of daily alcohol use exceeding the equivalent of four to five drinks per day by patient self-report [22], [23]. Indicators of social instability included recent or current homelessness, housing instability, familial dissolution, social isolation or incarceration.

The Batts-Ludwig system was used to assess fibrosis stage and inflammatory grade from liver biopsies [24], [25]. These measures were obtained from the biopsy taken closest to the first liver clinic visit, although biopsies taken more than five years before or one year following this visit were not considered. Cirrhosis was defined as either (i) stage 4 fibrosis on biopsy or (ii) a nodular liver contour plus at least one of three previously validated criteria: ascites, evidence of venous collateral vessels, or splenomegaly as visualized on CT scan, MRI, and/or ultrasound [26].

Patients were categorized into four HCV treatment-related groups using previously described standard definitions: SVR, relapsers, nonresponders, and never treated [6], [27], [28]. Relapsers achieved undetectable viral load during treatment with detectable virus found during a six month follow-up period. Nonresponders were null and partial responders who were detectably viremic throughout therapy. Those treated for <12 weeks were designated “early treatment discontinuation” (ETD) patients. Patients who were treated more than once were assigned the treatment category corresponding to their last course of therapy.

### Major Predictor and Outcome Measures

The primary outcome variables were time-to-cirrhosis and time-to-all-cause-death during the follow-up period. Never treated patients comprised the reference category. Time zero for all time-to-event analyses was the date of first liver clinic visit. To overcome the temporal bias associated with variations in treatment start time, we constructed a time-dependent covariate, using standard methods as previously described [29], [30]. This covariate modifies the major predictor (treatment outcome) by adjusting for differences in waiting times between time zero and the beginning of treatment. Time-to-cirrhosis was calculated from time zero to the date cirrhosis was first diagnosed or to the date of last liver clinic visit. Patients diagnosed with cirrhosis prior to time zero were excluded from the time-to-cirrhosis analysis, but were included in time-to-death analysis. For time-to-death analysis, the study length extended from time zero to the date of either death or liver transplantation. In surviving patients, the right censoring time was the date of last medical service encounter or six months before the date that the SSDI was searched, whichever was later. As the SSDI only includes date but not cause of death, all-cause death was used as the outcome variable. March 31, 2012 was the cut-off date for all observations.

### Statistical Methods

All analyses were performed using SAS version 9.2 (SAS Institute Inc., Cary, NC). Chi square tests were performed for categorical data analysis, and the Student’s t test or Wilcoxon rank sum test was used to evaluate the association of continuous predictors on categorical dependent variables (such as patient treatment group). Cox proportional hazards models were used to analyze both univariate and multivariate effects on the outcomes of interest. Since differences in baseline fibrosis stage likely have nonlinear influences on long-term fibrosis progression, and because the distribution of fibrosis stage violated the proportional hazards assumption, all Cox proportional hazards models were stratified by fibrosis stage groupings (0–1 and 2–3 in time-to-cirrhosis analyses and 0–1, 2–3 and 4 in time-to-death analysis). Time-to-cirrhosis analysis was repeated using an alternate fibrosis stratification strategy to allow a closer examination of advanced stage 3 compared to stages 0–2.

We used two strategies to adjust for the non-random distribution of characteristics differentiating treated from untreated patients, including age at initial liver biopsy, race/ethnicity, HCV genotype, history of heavy alcohol use, other substance use, psychiatric comorbidities, and social stability. First, these factors were assessed individually in univariate hazards models and incorporated into the full multivariate model through backward stepwise regression, as described in Statistical Methods S1. Second, propensity scores were derived from non-collinear risk factors and substituted into the final time-to-event models using previously described methods to estimate a composite effect from the factors related to treatment selection [31], [32]. Adjusted hazard ratios resulting from the two approaches were compared. After stratification by fibrosis stage, age-adjusted proportional hazards curves were generated to graph the hazard function for cirrhosis and death or liver transplantation among the four treatment outcome groups. These models assume proportional hazards for age, but not for treatment group.

## Results

### Demographic and Clinical Characteristics

Among SFVA patients screened, 358 (99% male) met the study inclusion criteria, and 159 patients (44.4%) received antiviral treatment for HCV. Approximately 80% were between the ages of 45 and 65 at initial liver clinic visit, with a mean age of 51 (Table 1). This age distribution corresponds to the birth cohort of Vietnam era veterans, a risk group known to have higher rates of prior IDU and HCV seroprevalence than other groups of veterans, which reflects likely exposure to HCV during and shortly after their service years in the 1960s and 1970s [33] [34]. Approximately 55% of the cohort had little or no liver disease at baseline, as measured by Batts-Ludwig fibrosis score, while 7.3% were cirrhotic. Mean follow-up time was 10 years, and 22% (n = 78) died during follow-up. One patient underwent liver transplantation. Deaths occurred among 8.7% of patients achieving SVR, 18.2% of relapsers, 28.6% of nonresponders, and 23.8% of the never treated patients (*p*<0.01).

**Table 1:** Demographic and Clinical Characteristics of the SFVA HCV Cohort.

Variable	Total	SVR	NR	Relapser	ETD/Unknown	No Treatment	p-value
	(N = 358)	(N = 69)	(N = 49)	(N = 22)	(N = 19)	(N = 199)	
**Age at 1st Liver Clinic Visit (Yr), Mean (SD)**	50.98 (6.68)	49.13 (6.87)	50.38 (5.18)	51.06 (4.82)	50.47 (7.68)	51.81 (6.92)	0.20**
**Male Gender**	354 (98.9%)	66 (95.7%)	49 (100.0%)	22 (100.0%)	19 (100.0%)	198 (99.5%)	0.16*
**Race/Ethnicity**							
Caucasian	236 (66.1%)	59 (85.5%)	31 (64.6%)	18 (81.8%)	10 (52.6%)	118 (59.3%)	0.01
African-American	72 (20.2%)	4 (5.8%)	11 (22.9%)	3 (13.6%)	3 (15.8%)	51 (25.6%)	
Latino	31 (8.7%)	3 (4.3%)	4 (8.3%)	1 (4.5%)	4 (21.1%)	19 (9.5%)	
Asian/API/Native American	18 (5.0%)	3 (4.3%)	2 (4.2%)	0 (0.0%)	2 (10.5%)	11 (5.5%)	
**HCV Genotype** †							
Genotype 1	246 (68.7%)	33 (47.8%)	40 (81.6%)	13 (59.1%)	10 (52.6%)	150 (75.4%)	<0.0001
Genotype 2	52 (14.5%)	16 (23.2%)	3 (6.1%)	5 (22.7%)	3 (15.8%)	25 (12.6%)	
Genotype 3	30 (8.4%)	10 (14.5%)	5 (10.2%)	3 (13.6%)	1 (5.3%)	11 (5.5%)	
Genotype 4	6 (1.7%)	0 (0.0%)	0 (0.0%)	0 (0.0%)	1 (5.3%)	5 (2.5%)	
Mixed genotype	2 (0.6%)	0 (0.0%)	1 (2.0%)	1 (4.5%)	0 (0.0%)	0 (0.0%)	
**Baseline Fibrosis Stage**							
0	111 (31.0%)	13 (18.8%)	5 (10.2%)	7 (31.8%)	2 (10.5%)	84 (42.2%)	<0.0001
1	87 (24.3%)	20 (29.0%)	10 (20.4%)	2 (9.1%)	5 (26.3%)	50 (25.1%)	
2	92 (25.7%)	26 (37.7%)	13 (26.5%)	5 (22.7%)	3 (15.8%)	45 (22.6%)	
3	42 (11.7%)	7 (10.1%)	13 (26.5%)	4 (18.2%)	6 (31.6%)	12 (6.0%)	
4	26 (7.3%)	3 (4.3%)	8 (16.3%)	4 (18.2%)	3 (15.8%)	8 (4.0%)	
**Baseline Inflammation Grade** ‡							
0	17(4.9%)	1(1.5%)	0(0.0%)	0(0.0%)	2(11.1%)	14(7.3%)	0.003
1	107(31.0%)	17(25.0%)	9(20.0%)	6(28.6%)	2(11.1%)	73(37.8%)	
2	195(56.5%)	42(61.8%)	33(73.3%)	11(52.4%)	14(77.8%)	95(49.2%)	
3	26(7.5%)	8(11.8%)	3(6.7%)	4(19.0%)	0(0.0%)	11(5.7%)	
**Baseline ALT, Mean (SD)**	94.01 (71.91)	109.84 (74.59)	92.89 (71.06)	99.69 (75.38)	76.47 (65.28)	89.17 (71.56)	0.09**
**Baseline BMI, Mean (SD)**	28.42 (5.25)	29.86 (5.10)	29.04 (5.23)	28.20 (4.71)	28.74 (7.62)	27.75 (5.02)	0.02**
**Diabetes Mellitus**	56 (15.6%)	9 (13.0%)	10 (20.4%)	5 (22.7%)	4 (21.1%)	28 (14.1%)	0.50*
**Blood Transfusion before 1992**	71 (21.1%)	14 (22.2%)	8(17.8%)	6 (30.0%)	3 (15.8%)	40 (21.2%)	0.82*
**Number of Follow-up Images/Liver Biopsy, Mean (SD)**	2.10 (1.96)	1.77 (2.09)	3.88 (2.25)	2.77 (1.63)	1.68 (1.83)	1.74 (1.62)	<0.0001**
**Cirrhosis during Follow-up**	60 (18.1%)	7 (10.6%)	20 (48.8%)	4 (22.2%)	2 (12.5%)	27 (14.1%)	<0.0001*
**HCC during Follow-up**	20 (5.6%)	2 (2.9%)	6 (12.2%)	1 (4.5%)	2 (10.5%)	9 (4.5%)	0.14*
**Liver Transplant during Follow-up**	1 (0.3%)	0 (0.0%)	0 (0.0%)	0 (0.0%)	1 (5.3%)	0 (0.0%)	0.05*
**Died during Follow-up**	78 (21.8%)	6 (8.7%)	14 (28.6%)	4 (18.2%)	7 (36.8%)	47 (23.6%)	0.01*
**Years of Follow-Up, Mean (SD)**	10.00 (3.05)	10.76 (2.88)	11.72 (2.89)	10.46 (3.12)	8.97 (4.27)	9.36 (2.78)	<0.0001**
**Treated Patients Only**	**N = 159**						
**Courses of IFNα Treatment**							
1	133 (83.6%)	62 (89.9%)	36 (73.5%)	19 (86.4%)	16 (84.2%)	NA	0.13*
≥2	26 (16.4%)	7 (10.1%)	13 (26.5%)	3 (13.6%)	3 (15.8%)	NA	
**Length of IFNα treatment (wk), Mean (SD)**	40.45 (22.32)	43.38 (17.11)	40.00 (22.27)	51.18 (27.68)	18.53 (19.25)	NA	<0.0001**
**Therapeutic Regimen**							
IFNα monotherapy	20 (12.6%)	6 (8.7%)	9 (18.4%)	2 (9.1%)	3 (15.8%)	NA	0.41*
IFNα/RBV therapy	139 (87.4%)	63 (91.3%)	40 (81.6%)	20 (90.9%)	16 (84.2%)	NA	

P-values were calculated from Chi-square test for category variables and ANOVA for continuous variables unless otherwise marked.

* P-values were calculated from Fisher’s Exact test.

** Variable was rank transformed.

† 22 cases missing HCV genotype data.

‡ 13 cases missing baseline inflammation grade.

Treated SFVA patients had higher liver fibrosis stage and inflammation score at baseline, however when stratified by fibrosis stage the influence of inflammation lost significance, suggesting effect modification by fibrosis. Treated patients were followed for a longer period than never treated patients (10.8 versus 9.4 years, *p*<0.0001) and treatment nonresponders and relapsers had more follow-up liver imaging (or liver biopsies) than never treated patients (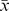 = 1.77, 2.77, 3.88 and 1.74 for SVR, relapsers, nonresponders and never treated patients, respectively, *p*<0.0001) (Table 1). Never treated patients were older (52 versus 50 years, *p* = 0.04), more likely to be African American and more likely to be infected with “difficult to clear” genotypes HCV genotypes 1 or 4 than 2 or 3 (Table 1). Treated patients had a higher mean BMI at baseline than never treated patients (Table 1). The most common reasons cited for the decision not to treat during follow-up were minimal liver disease (21.6%), ongoing alcohol and substance use (19.1%), active mental health problems (11.1%), African American race (5.0%), advanced liver disease (3.0%), and advanced age with or without other comorbidities (8.5%). Compared with treated patients, never treated patients were more likely to be active substance users and to have at least one indicator of social instability (Table 2).

**Table 2:** Risk Factors Characterizing Treated and Untreated Patient Groups (SFVA Cohort).

Variable	Total	SVR	NR	Relapse	ETD/Unknown	No Treatment	p-value
	(N = 358)	(N = 69)	(N = 49)	(N = 22)	(N = 19)	(N = 199)	
**History of Heavy Drinking**	224 (62.6%)	35 (50.7%)	33 (67.3%)	13 (59.1%)	12 (63.2%)	131 (65.8%)	0.23
**Active IDU**	19 (5.3%)	1 (1.5%)	1 (2.0%)	0 (0.0%)	1 (5.3%)	16 (8.1%)	0.16*
**Active Substance Use (non-IDU)**	60 (17.2%)	6 (9.2%)	5 (10.9%)	4 (18.2%)	1 (5.3%)	44 (22.4%)	0.05*
**Current Methadone**	44 (12.4%)	5 (7.4%)	9 (18.4%)	1 (4.5%)	3 (15.8%)	26 (13.2%)	0.31*
**History of Depression**	133 (38.0%)	27 (39.7%)	24 (50.0%)	9 (42.9%)	8 (42.1%)	65 (33.5%)	0.45
**PTSD**	59 (16.9%)	14 (20.6%)	12 (24.5%)	4 (19.0%)	2 (10.5%)	27 (14.0%)	0.43
**Social Stability**	307 (86.5%)	67 (97.1%)	47 (97.9%)	20 (90.9%)	19 (100.0%)	154 (78.2%)	<0.0001

* P-values were calculated from Fisher’s Exact test.

No statistically significant difference was found in mean baseline ALT between treated and never treated groups (*p* = 0.15, data not shown), although the SVR group had a marginally higher baseline value as compared to the other treatment outcome groups (Table 1). We compared the mean change in ALT before and after treatment for nonresponders and relapsers (grouped together) to that of never treated patients for a comparable time interval. We found that nonresponders and relapsers had a mean decrease in ALT of 15.0 U/L, while never treated patients had a mean increase of ALT of 17.6 U/L (*p* = 0.05) (data not shown). In further analysis we found that 69.2% of nonresponders and relapsers had a decrease in ALT ≥25% following treatment as compared to 45.2% of never treated patients, while 19.2% of nonresponders and relapsers and 41.9% of never treated patients had an increase in ALT of ≥25% (*p = *0.03) (data not shown).

#### Predictors of treatment success

Among treated SFVA patients, African Americans were less likely to achieve SVR than other races/ethnic groups (*p* = 0.03) as were patients with HCV genotypes 1 or 4 (*p* = 0.007) and those with higher pretreatment fibrosis stage (*p* = 0.004). No significant difference was noted in treatment success between the small number of SFVA patients who received IFNα monotherapy and those treated with IFNα or pegylated IFNα/RBV combination therapy (Table 1).

#### Cumulative incidence of cirrhosis

**Figure 1:**
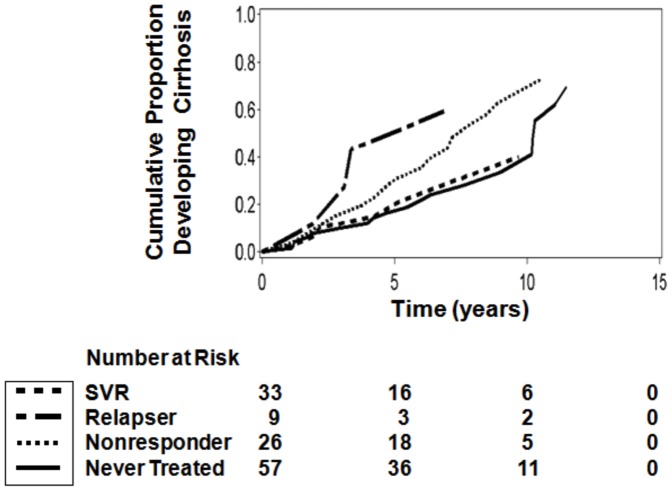
Cumulative incidence of cirrhosis among SFVA patients with baseline fibrosis stages 2 and 3. Age adjusted proportional hazards curves indicate that treatment relapsers and nonresponders had a higher incidence of cirrhosis over the study period compared to never treated patients, but these differences were not statistically significant unadjusted for other risk factors.

We examined the cumulative incidence of cirrhosis among SFVA patients with baseline fibrosis stages 2 and 3 using age adjusted proportional hazards curves stratified by treatment group (Figure 1). A greater proportion of treatment relapsers and nonresponders developed cirrhosis than never treated patients. The overall incidence of cirrhosis in the SFVA cohort was 25.8 cases per 1,000 person years. While SVR and never treated patients had incidence rates of 16.2 and 20.5 cases per 1,000 person years, respectively, these rates rose to 28.9 and 58.9 cases per 1,000 person years among relapsers and nonresponders. These differences, however, were not statistically significant.

#### Time-to-cirrhosis analysis

In univariate proportional hazards models stratified by baseline fibrosis stage (0–1 and 2–3) and employing the time dependent covariate for SFVA treatment group, nonresponders were twice as likely to develop cirrhosis when compared to never treated patients (HR = 2.02, CI 1.11–3.67, Table 3). Patients achieving SVR did not realize appreciable protection from cirrhosis in these models, although their hazard ratios trended in that direction. Histological inflammation score was not predictive of cirrhosis once the cohort was stratified by baseline fibrosis stage. As expected, age incrementally increased the cirrhosis risk; for every additional year there was a 5% increase in the hazard of developing cirrhosis (HR, 1.05 CI 1.01–1.09). History of blood transfusion prior to 1992 was also associated with an increased the hazard of cirrhosis (HR 2.04, CI 1.16–3.59). African American patients were at considerably lower risk of developing cirrhosis than were Caucasians (HR = 0.47, CI 0.20–1.10), but this difference did not achieve statistical significance, probably due to the low number of cirrhosis events among African Americans. In contrast, Latinos were at greater risk of cirrhosis compared to Caucasians, but again this increased hazard did not attain statistical significance (HR = 1.82, CI 0.88–3.77). Neither BMI, diabetes mellitus, history of heavy alcohol use or lack of social stability were significantly associated with the hazard of cirrhosis in univariate models. ALT was not entered into the model as it was not a significant predictor of the hazard of cirrhosis.

**Table 3:** Predictors of Development of Cirrhosis Stratified by Fibrosis Stage (SFVA Cohort, N = 332)*.

Characteristics‡	Univariate Model	Multivariate Model
	HR† (95% CI)	HR† (95% CI)
**Treatment outcome**		
SVR	0.67 (0.23–1.56)	0.68 (0.26–1.80)
Nonresponder	2.02 (1.11–3.67)	2.35 (1.18–4.69)
Relapser	1.24 (0.43–3.55)	1.00 (0.28–3.56)
ETD or Lost to Follow–up	1.07 (0.25–4.52)	1.28 (0.29–5.69)
Never Treated	ref	
**Age at first liver clinic visit–per year increase** §	1.05 (1.01–1.09)	1.09 (1.04–1.14)
**Race** §		
African American	0.61 (0.27–1.36)	0.30 (0.13–0.72)
Latino	1.82 (0.88–3.77)	2.50 (1.12–5.56)
Asian/API/Native American	0.41 (0.06–3.09)	0.42 (0.06–3.17)
Caucasian	ref	Ref
**HCV genotype 1 or 4** §	1.40 (0.77–2.56)	2.33 (1.10–4.93)
**BMI-per unit increase** §	1.03 (0.98–1.08)	1.07 (1.02–1.13)
**Active Substance Use (non–IDU)** §	1.10 (0.56–2.18)	NA
**Social Stability** §	0.72 (0.35–1.46)	0.48 (0.21–1.09)
**Transfusion before 1992**	2.04 (1.16–3.59)	NA

* Cox Proportional Hazards Models using time dependent covariate correcting for differences in waiting times from baseline to treatment initiation.

† Hazard Ratio (HR) calculated using Cox Proportional Hazards Modeling.

‡ Interaction terms not shown.

§ Risk factors that significantly differentiate the treated from never treated groups.

Results of multivariate proportional hazards analysis stratified by pretreatment fibrosis stage in the SFVA cohort are presented in Table 3. The increased hazard of progression to cirrhosis among treatment nonresponders relative to never treated patients remained significant in this model, after adjustment for demographic and clinical characteristics and the factors that differentiated treated from never treated groups (HR = 2.35, CI 1.18–4.69). Latino ethnicity now became significantly associated with cirrhosis (HR = 2.50, CI 1.12–5.56), while African American race was protective (HR = 0.30, CI 0.13–0.72). Age continued to have an incremental effect as did BMI. Patients with HCV genotypes 1 or 4 were at increased risk of cirrhosis, even after correcting for the interaction between genotype and treatment initiation. Restratifying to compare more advanced baseline fibrosis stage 3 against stages 0–2 and repeating these analyses, we found that treatment nonresponders continued to exhibit an increased hazard of cirrhosis compared to never treated patients (HR = 2.95, CI 1.34–6.52, in the multivariate model, data not shown).

Since fibrosis progression is thought to proceed more slowly in African Americans compared to Caucasian patients with chronic HCV, we attempted to reanalyze these data separately for African American and non-African American patient groups [35], [36], [37]. There were too few cirrhosis events to develop a proportional hazards model for cirrhosis development using the multivariate modeling strategy described above, however when using a pre-fitted model, we found that treatment non-response (HR = 2.05, CI 0.99–4.26), age (HR = 1.07, CI 1.02–1.12) and BMI (HR = 1.08, CI 1.02–1.15) were each significant predictors of an increased hazard of cirrhosis among African Americans (data not shown).

Finally, as an alternative strategy to account for the inherent differences between treated and never treated groups, we substituted a propensity score for statistically significant psychosocial risk factors in our multivariate proportional hazards models as described in Statistical Methods S1. These results differed little from the findings above, and again treatment nonresponders had a significantly increased hazard of cirrhosis compared to never treated patients (HR 2.68, CI 1.27–5.63, data not shown).

### Replication Cohort

In order to confirm these unexpected findings, we used identical data collection and analytic methods to replicate the study in an independent cohort of patients with chronic HCV from the UCSF Liver Clinic (N = 265, Replication Cohort S1, Table S1). These patients were subject to the same eligibility criteria as the SFVA patients. All UCSF patients had a baseline liver biopsy and follow-up liver imaging or biopsy. UCSF cohort patients were significantly younger than the SFVA group (baseline age 48 versus 51 years for the SFVA) and included 46% female patients, whereas the SFVA group was 99% male (Table 4). There was a significantly higher proportion of Caucasian and Asian patients and a lower proportion of African American and Latino patients in the UCSF cohort (*p*<0.0001). The mean follow-up time was 7.7 years as compared to 10 years for the SFVA (*p*<0.0001) (Table 4). Descriptive characteristics of the UCSF cohort are provided in Tables S1 and S2.

**Table 4:** Selected Characteristics of SFVA and UCSF HCV Cohorts.

Variable	SFVA	UCSF	p-value
	(N = 358)	(N = 265)	
**Age at 1st Liver Clinic Visit (Yr), Mean (SD)**	50.98 (6.68)	48.42 (8.39)	<0.0001*
**Gender**			
Female	4 (1.1%)	123 (46.4%)	<0.0001
Male	354 (98.9%)	142 (53.6%)	
**Ethnicity**			
Caucasian	236 (66.1%)	186 (72.4%)	<0.0001
African-American	72 (20.2%)	21 (8.2%)	
Latino	31 (8.7%)	11 (4.3%)	
Asian/API/Native American	18 (5.0%)	39 (15.2%)	
**Cirrhosis during Follow-up**	60 (18.1%)	28 (11.7%)	0.04
**Death**	78 (21.8%)	27 (10.2%)	0.0001
**Liver Transplant**	1 (0.3%)	12 (4.5%)	0.0002
**Years of Follow-up, Mean (SD)**	10.00 (3.05)	7.55 (4.09)	<0.0001*
**Treatment Groups**			
Never treated	199 (55.6%)	134 (50.6%)	0.21
Treated	159 (44.4%)	131 (49.4%)	
**Treated Patients Only** †			
**Length of all IFNα treatment (wk), Mean (SD)**	40.45 (22.32)	44.82 (36.13)	0.77*
**Treatment Outcome**			
Nonresponder	49 (30.8%)	42 (32.1%)	0.17
Relapser	22 (13.8%)	21 (16.0%)	
ETD or Lost to Follow-up	19 (11.9%)	25 (19.1%)	
SVR	69 (43.4%)	43 (32.8%)	

P-values were calculated from Chi-square test for category variables and ANOVA for continuous variables unless otherwise marked.

* Variable was rank transformed.

† Including 159 treated patients at VA and 131 treated patients at Moffitt.

Cumulative incidence of cirrhosis among UCSF patients with baseline fibrosis stages 2 and 3 was examined using age adjusted proportional hazards curves (Figure S1), which indicate that there were no differences in the incidence of cirrhosis among treatment groups, when unadjusted for other risk factors. In univariate proportional hazards models stratified by baseline liver fibrosis stage (0–1 and 2–3), UCSF treatment nonresponders showed an increased hazard for developing cirrhosis when compared to the never treated group, but this result fell just short of statistical significance (HR = 2.28, CI 0.93–5.59, Table S3). SVR was not significantly protective against cirrhosis. In multivariate proportional hazards models stratified by fibrosis stages (0–1 and 2–3) and adjusted for other risk factors, treatment nonresponse independently increased the long-term hazard of cirrhosis (HR = 5.90, CI 1.50–23.24, Table S3). Consistent with the SFVA cohort, incremental increases in age and BMI also contributed to the hazard of cirrhosis. In this model, social stability was significantly protective against cirrhosis (HR = 0.23, CI 0.07–0.79, Table S3). Restratifying to compare patients with advanced baseline fibrosis stage 3 to those with stages 0–2 still resulted in a significantly increased hazard of progression to cirrhosis among treatment non-responders in the final multivariate model (HR = 4.30, CI 1.16–15.93, data not shown).

### Survival

**Figure 2:**
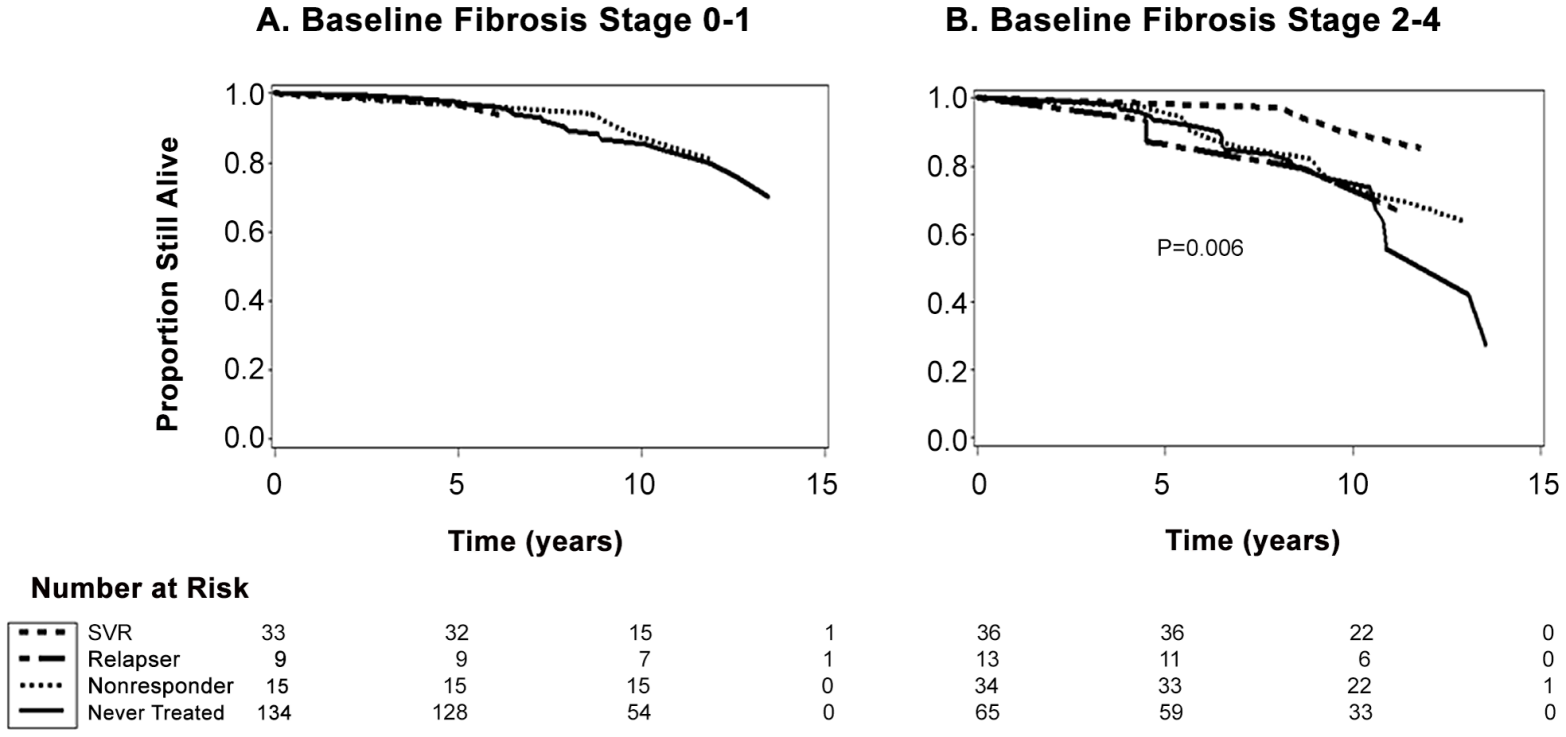
Proportion remaining alive among SFVA patients with baseline liver fibrosis stage 0–1 (panel A) and stage 2–4 (panel B). Age adjusted proportional hazards curves indicate that there were no significant differences in survival among the four treatment groups in patients with fibrosis stage 0–1 (panel A). Among patients with fibrosis stage 2–4, SVR significantly improved survival compared to never treated patients (p = 0.006), unadjusted for other risk factors (panel B).

Figure 2 depicts the survival experience for SFVA patients in age adjusted proportional hazards curves for two groups defined by baseline liver fibrosis stage. No differences in survival were observed among treatment groups for patients with baseline fibrosis stage 0–1 (Figure 2, panel A), whereas SVR led to a significant survival benefit compared to never treated patients among those with baseline fibrosis stage 2–4 (*p* = 0.006) (Figure 2, panel B).

Cox proportional hazards models were developed to examine the contribution of multiple risk factors on time-to-death (Table 5). Time-to-death models were stratified by baseline fibrosis stage 0–1, 2–3, and 4, and incorporated the time dependent covariate described above. In univariate analysis of the SFVA cohort, both SVR and nonresponder patients had a lower hazard of dying compared with never treated patients (HR = 0.24, CI 0.10–0.58 and HR = 0.51, CI 0.26–0.98, respectively, Table 5). The hazard ratio for treatment relapsers also tended toward protection, but did not achieve significance. Older age at baseline liver biopsy, Latino ethnicity, heavy alcohol use, and prior blood transfusion each significantly increased the hazard of death during follow-up in univariate models (Table 5). After adjusting for psychosocial and clinical risk factors in multivariate proportional hazards analysis, SVR patients and relapsers each had a significant survival advantage (HR = 0.23, CI 0.07–0.75 and HR = 0.11, CI 0.01–0.95, respectively) compared to never treated patients. While the hazard ratio for nonresponders tended toward protection, it did not achieve statistical significance. Substituting propensity scores for individual psychosocial risk factors did not affect the directionality or significance of the hazard ratios for the major predictors in time-to-death analysis (data not shown).

**Table 5:** Predictors of Death or Liver Transplant Stratified by Fibrosis Stage (SFVA Cohort, N = 358)*.

Characteristics‡	Univariate Model	Multivariate Model
	HR† (95% CI)	HR† (95% CI)
**Treatment Outcome**		
SVR	0.24 (0.10–0.58)	0.23 (0.07–0.75)
Nonresponder	0.51 (0.26–0.98)	0.56 (0.24–1.32)
Relapser	0.48 (0.17–1.35)	0.11 (0.01–0.95)
ETD or Lost to Follow-up	1.32 (0.59–2.94)	1.44 (0.52–4.03)
Never Treated	ref	Ref
**Age at 1st liver clinic visit-per year increase**	1.08 (1.05–1.12)	1.1 (1.06–1.15)
**Race**		
African American	1.07 (0.59–1.97)	0.43 (0.20–0.93)
Latino	1.89 (1.00–3.57)	1.73 (0.74–4.02)
Asian/API/Native American	0.25 (0.03–1.82)	0.5 (0.07–3.77)
Caucasian	ref	Ref
**BMI–per unit increase**	1.02 (0.97–1.06)	1.07 (1.02–1.12)
**History of Active Alcohol Abuse**	1.67 (1.01–2.75)	1.82 (0.99–3.35)
**Transfusion before 1992**	1.66 (0.97–2.82)	3.36 (1.20–9.44)

* Cox Proportional Hazards Models.

† Hazard Ratio (HR) calculated using Cox Proportional Hazards Modeling.

‡ Interaction terms not shown.

Time-to-death or liver transplantation analyses were repeated for the UCSF cohort. Proportionally fewer UCSF patients died and more underwent liver transplantation than SFVA patients (Table 4). We examined the proportion remaining alive among UCSF patients with baseline liver fibrosis stage 0–1 and stage 2–4 in age adjusted proportional hazards curves (Figure S2, panels A and B, respectively). There were no deaths among treated patients with baseline fibrosis stage 0–1 (panel A). Among patients with baseline fibrosis stage 2–4, a greater proportion of relapsers and never treated patients died during follow-up compared with either SVR or nonresponder patients, but these differences were not statistically significant (panel B). In univariate proportional hazards models, stratified by baseline fibrosis stage, patients achieving SVR had a marginally significant reduction in death compared to never treated patients (HR = 0.24, CI 0.05–1.06, Table S4). This advantage did not extend to either nonresponders or relapsers. Incremental increases in baseline age were also contributory (HR = 1.07, CI 1.02–1.12, Table S4). In multivariate survival analysis, however, no risk factors achieved statistical significance.

## Discussion

The present study measured long-term outcomes in patients with chronic HCV in two independent cohorts followed over the course of 7.7 to 10 years. Cohort patients were heterogeneous with regard to demographic and psychosocial characteristics, representing typical clinical practice, and data collection methods were optimized to maximize validity and measure known confounders. Unlike previously published studies, SVR was not associated with significant protection against cirrhosis in either cohort, even after stratifying for baseline levels of liver fibrosis and adjusting for liver inflammation [16], [17], [38], [39], [40]. Surprisingly, we found that the hazard of cirrhosis among treatment nonresponders was more than twice that of never treated patients in both cohorts. These results persisted after adjustment for clinical and psychosocial risk factors using two alternative adjustment strategies. Also, unlike previous studies, neither baseline ALT level nor change in ALT before and after completion of treatment was associated with progression to cirrhosis.

Although our study is not intended to identify an explanatory mechanism for this finding, it raises the question of whether hepatic inflammation and fibrosis could be increased by immunostimulatory IFNα-based antiviral therapies in cases where HCV is not eradicated. IFNα/RBV can trigger broad and robust antiviral T cell responses, which are beneficial when they result in SVR, but might contribute to worsened inflammation and scarring (cirrhosis) in the continued presence of viral antigens [41], [42], [43], [44]. Lower rates of both cirrhosis and SVR among African Americans illustrate the point that lower inflammatory responses may be favorable in certain circumstances [35], [37]. Further research is needed to explore this possibility.

The long-term effects of IFNα-based anti-HCV treatment on liver disease progression in noncirrhotic patients have been difficult to quantify from previous studies. In a meta-analysis of HCV cohort studies with greater than one year of follow-up, nearly 70% tracked subjects for less than seven years, whereas the mean duration of follow-up among patients in our SFVA cohort was 10 years [40]. Few previous studies have specifically compared the experience of treated patients to those who were never treated, and none specifically explored the hypothesis that failed IFNα-based treatment could increase the long-term risk of cirrhosis [38], [45].

In recent years, there has been an emphasis on studies of IFNa-based retreatment in previous nonresponders and relapsers and their outcomes compared to those achieving SVR [46], [47]. The most notable of these were the HALT-C and EPIC^3^ trials which enrolled previously treated patients with advanced fibrosis. These two prospective studies examined histologic effects of low dose maintenance pegylated IFNα in prior HCV treatment failures with METAVIR F2 and F3 fibrosis at study initiation [48]. Patients were randomized to low dose maintenance pegylated IFNα therapy or observation and assessed for fibrosis response using repeat liver biopsies after a mean interval of 3.7 years. Results from the EPIC^3^ study showed no statistically significant differences between METAVIR fibrosis scores of the treated and observation groups at the end of the study period [48]. HALT-C investigators extended the study for up to an 8 year period of observation and found that the annual rate of initial liver-related complications was higher among the pegylated IFNα group than among the controls [49]. Moreover, histologic features on sequential liver biopsies led the HALT-C investigators to speculate that pegylated IFNα might be associated with a long-term worsening of liver related morbidity in treatment nonresponders and in excess mortality among those with advanced liver disease [50].

In one of the few large-scale studies to compare outcomes between IFNα treated and untreated patients, the Japanese IHIT Study Group followed patients who had been previously treated with IFNα monotherapy over a median period of 3.7 years, using paired biopsies to compare liver fibrosis progression among SVR patients, patients without SVR and untreated patients, stratified by fibrosis stage at initial biopsy [51], [52]. Among patients with initial METAVIR F2 or F3 fibrosis, a *post hoc* analysis of primary data presented in this report found no significant difference in cirrhosis development among patients without SVR and untreated patients [51]. None of the patients with initial F2 or F3 fibrosis who achieved SVR developed cirrhosis. These data suggest that failed therapy may not increase the risk of cirrhosis during intermediate (3–5 year) follow-up.

Unlike the patients in the Japanese cohort, the majority of our patients were treated with IFNα/RBV combination therapy, rather than with IFNα monotherapy, and our follow-up period was more than twice as long. Our study included few Asian patients, who have a higher probability of achieving SVR, but also are more likely to progress to cirrhosis [28]. Our finding that treatment failures have an increased long-term hazard of cirrhosis is thus neither directly supported nor contradicted by this or any other published report.

Previously published studies also found that never treated HCV patients had a greater mortality risk than patients who achieve SVR, and in some cases, those who fail treatment [38], [52]. In another IHIT study, Yoshida et al. found that the overall risk of death was reduced among IFNα treated patients, including treatment nonresponders, as compared to patients not receiving treatment [52]. Their multivariate Cox proportional hazards models were adjusted by gender, age and IFNα therapy outcome. When survival analysis was further stratified by cirrhotic and noncirrhotic patients, IFNα therapy was associated with improved survival among the noncirrhotic patients only [52]. A recent Cochrane Review of seven trials, including the HALT-C and EPIC^3^ studies, found a significant increase in all-cause mortality in IFNα maintenance patients and concluded that patients with severe fibrosis who failed previous IFNα treatment did not derive a survival benefit from further therapy with pegylated IFNα [46].

These studies did not assess the effects of clinical and behavioral risk factors on liver disease outcomes as comprehensively as ours did. Our multivariate time-to-death analyses reveal that, even though nonresponders had more than twice the hazard of cirrhosis, their survival was not significantly different from that of never treated patients. The effects of cirrhosis on survival in our cohort may be offset by the relatively younger age and more beneficial clinical and psychosocial risk factor profile of nonresponders compared with never treated patients. Our findings suggest that some previously reported benefits of therapy among treatment failures might be attributable to the lower concomitant risks associated with treatment candidacy, rather than to disease modifying benefits of pharmacologic therapy [38], [52].

As is the case in most nonrandomized studies, the presence of bias by indication can be difficult to resolve [53], [54]. We were especially concerned about confounding from risk factors that independently could promote the development of cirrhosis among patients with HCV and also influence the decision whether or not to initiate antiviral therapy. We carefully assessed an array of clinical and psychosocial risk factors and, not unexpectedly, found that never treated patients were older, more likely to engage in ongoing alcohol or other substance abuse, and to experience social instability compared with treatment nonresponders. We speculated that differing biobehavioral risk profiles were unlikely to account for the reduced incidence of cirrhosis in untreated patients since many of these would be predicted to increase, rather than reduce fibrosis progression. We used two alternative strategies to statistically adjust for these potential confounders in our Cox proportional hazards models [27]. Using either adjustment method, treatment nonresponders were found to have a significantly greater hazard of developing cirrhosis than the never treated group–a finding that was observed in the both the SFVA and UCSF cohorts. Some differences between treated and never treated patients were identified that could not be completely corrected in our statistical models. Treated patients were followed for approximately 1.5 years longer than never treated patients and had, on average, one additional diagnostic procedure. Both the duration of follow-up and the number of diagnostic procedures were entered into predictive models, but were not found to be confounders for either outcome (time-to-cirrhosis or time-to-death). It is still possible that unmeasured confounding factors linked to treatment failure may have biased the results, but rigorous data collection, robust statistical methods, and the stability of a significant hazard ratio for cirrhosis among nonresponders in both cohorts make a compelling case for the validity of these findings. Furthermore the congruence of the findings from two diverse patient populations–the SFVA cohort comprised of comparably aged veterans with similar risk behavior histories and the more demographically diverse UCSF cohort–suggest that these results may be generalizable.

Our results suggest the possibility that treatment with IFNα-based regimens without viral clearance may be associated with progressive liver disease. Although these data reflect the long-term outcomes for two entire patient cohorts at independent institutions, they should be interpreted with caution as they are derived from retrospective chart reviews. If confirmed, these results make a compelling case for the enhanced use of sensitive diagnostic and predictive tools, including recently described genetic tests, to identify patients most likely to benefit from IFNα-based treatment [36], [55]. Moreover, the potential for adverse outcomes should be considered in current and future studies examining HCV treatment using pegylated-IFNα/RBV in combination with newer agents such as HCV protease inhibitors, as a substantial proportion of null or partial responders with advanced fibrosis will emerge from these treatment groups. In particular, it may be advisable not to retreat these patients with IFNα, but to keep them under observation until IFNα-free regimens are available.

## Supporting Information

Figure S1 **Cumulative incidence of cirrhosis among UCSF patients with baseline fibrosis stages 2 and 3.** Age adjusted proportional hazards curves indicate that there were no differences in the incidence of cirrhosis among treatment groups, unadjusted for other risk factors.
(TIF)

Figure S2 Proportion remaining alive among UCSF patients with baseline liver fibrosis stage 0–1 (panel A) and stage 2–4 (panel B). Age adjusted proportional hazards curves indicate that there were no deaths among treated patients with baseline fibrosis stage 0–1 (panel A). Amongst patients with baseline fibrosis stage 2–4, relapsers and never treated a greater proportion died during follow-up than among SVR and nonresponders patients, but these differences were not statistically significant.
(TIF)

Table S1 **Demographic and Clinical Characteristics of the UCSF Cohort.**
(DOC)

Table S2 **Risk Factors of Treated and Untreated Patient Groups (UCSF Cohort).**
(DOC)

Table S3 **Predictors of Development of Cirrhosis Stratified by Fibrosis Stage (UCSF Cohort).**
(DOC)

Table S4 **Predictors of Death or Liver Transplant Stratified by Fibrosis Stage (UCSF Cohort).**
(DOC)

Statistical Methods S1 **Details of Construction and Reduction of Statistical Models.**
(DOC)

Replication Cohort S1 **UCSF Medical Center Liver Clinic.**
(DOC)
