# Supplementary figures and images for: Nonresponse to Interferon-α Based Treatment for Chronic Hepatitis C Infection Is Associated with Increased Hazard of Cirrhosis
Source: PLoS One. 2013 Apr 25;8(4):e61568. doi: 10.1371/journal.pone.0061568 (PMC3636226; doi:10.1371/journal.pone.0061568)

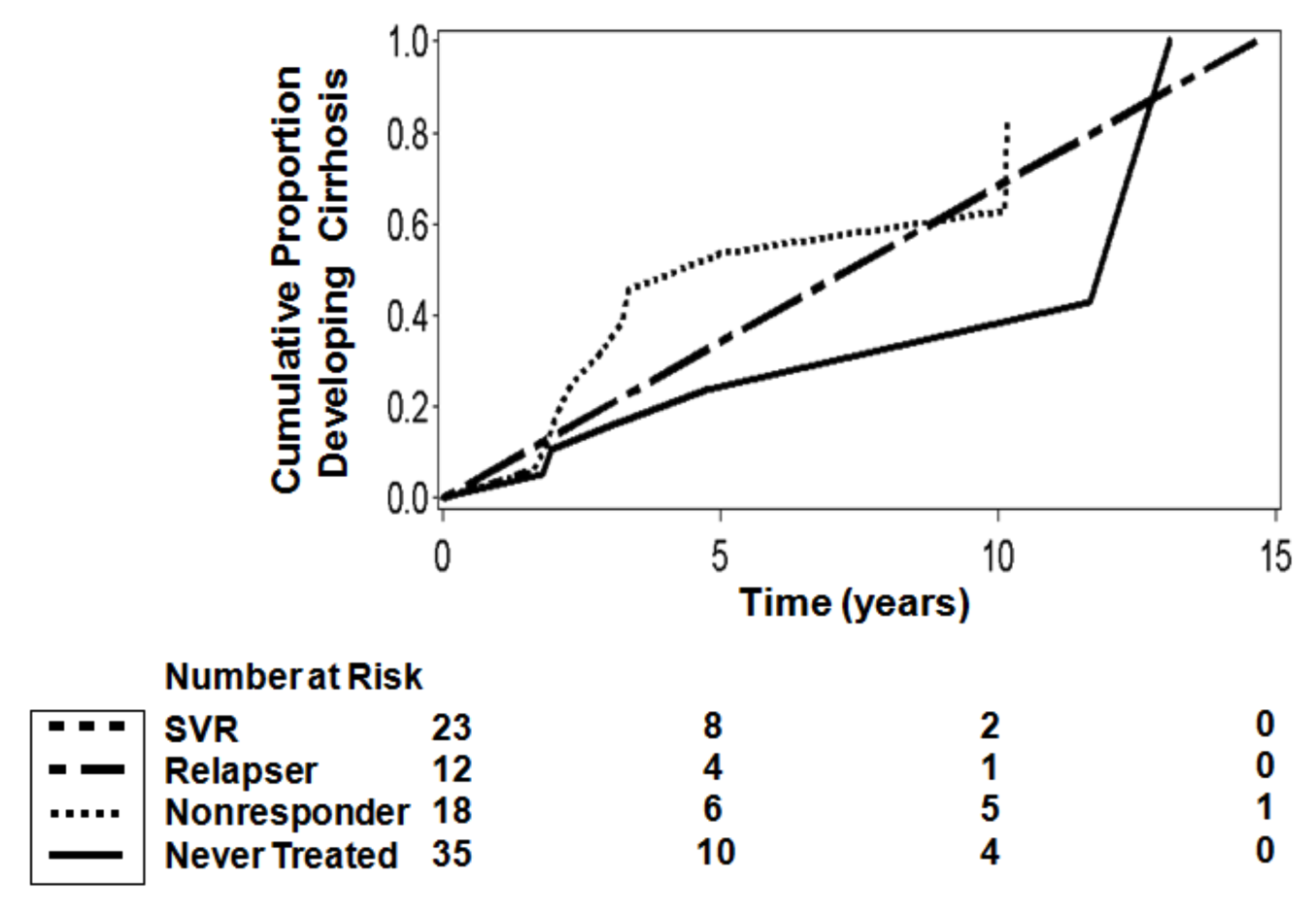

Supplement: Figure S1 — Cumulative incidence of cirrhosis among UCSF patients with baseline fibrosis stages 2 and 3. Age adjusted proportional hazards curves indicate that there were no differences in the incidence of cirrhosis among treatment groups, unadjusted for other risk factors. (TIF) [file pone.0061568.s001.tif]

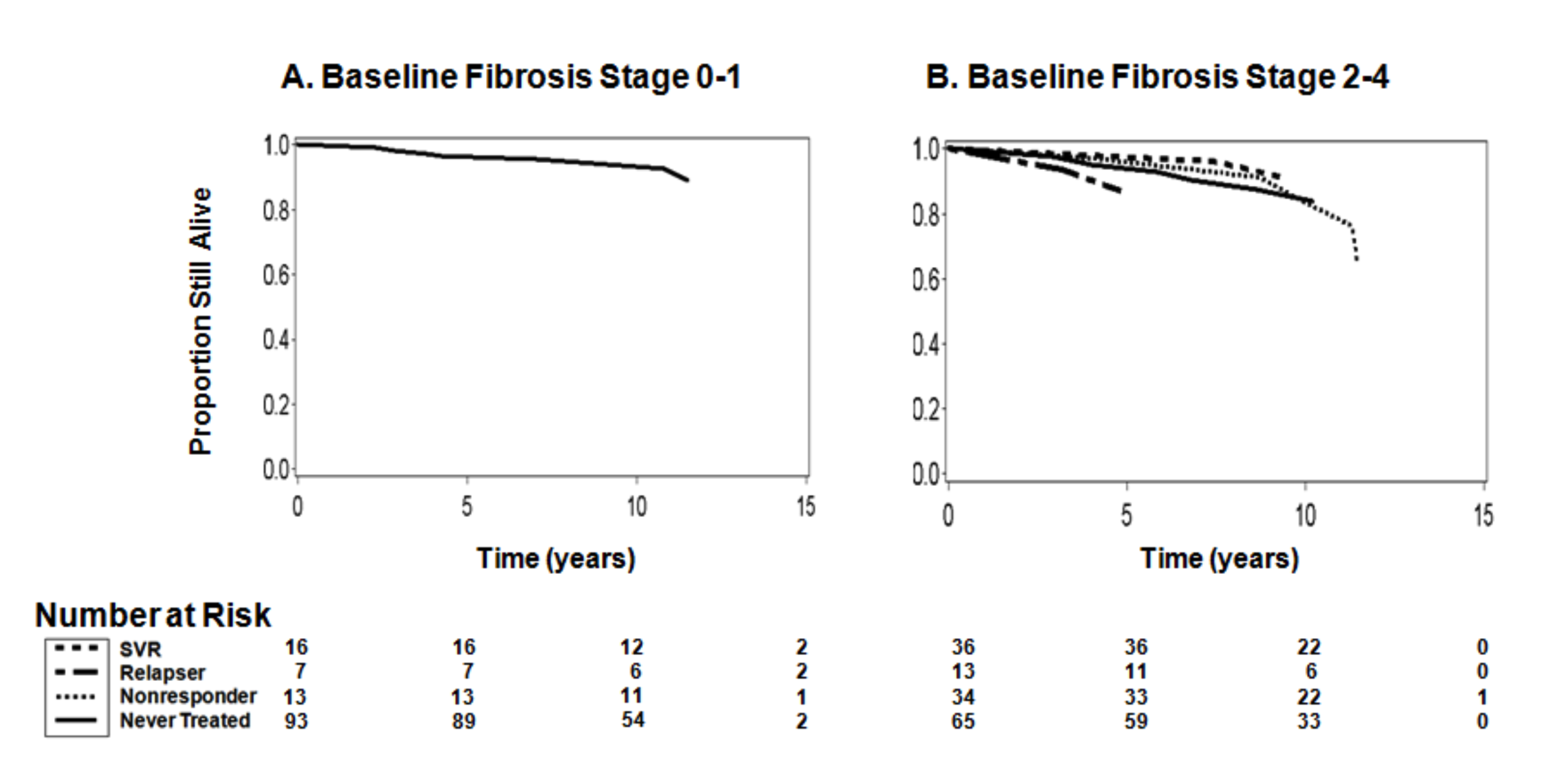

Supplement: Figure S2 — Proportion remaining alive among UCSF patients with baseline liver fibrosis stage 0–1 (panel A) and stage 2–4 (panel B). Age adjusted proportional hazards curves indicate that there were no deaths among treated patients with baseline fibrosis stage 0–1 (panel A). Amongst patients with baseline fibrosis stage 2–4, relapsers and never treated a greater proportion died during follow-up than among SVR and nonresponders patients, but these differences were not statistically significant. (TIF) [file pone.0061568.s002.tif]
