# Supplementary material for: Nonresponse to Interferon-α Based Treatment for Chronic Hepatitis C Infection Is Associated with Increased Hazard of Cirrhosis
Source: PLoS One. 2013 Apr 25;8(4):e61568. doi: 10.1371/journal.pone.0061568 (PMC3636226; doi:10.1371/journal.pone.0061568)
Supplement: Table S1 — Demographic and Clinical Characteristics of the UCSF Cohort. (DOC) [file pone.0061568.s003.doc]

**Table S1. Demographic and Clinical Characteristics of the UCSF Cohort**

| **Variable** | **Total** | **SVR** | **NR** | **Relapser** | **ETD** | **No Treatment** | **p-value** |
| --- | --- | --- | --- | --- | --- | --- | --- |
|  | **(N=265)** | **(N=43)** | **(N=42)** | **(N=21)** | **(N=25)** | **(N=134)** |  |
| **Age at 1st Liver Clinic Visit (Yr), Mean (SD)** | 48.42 (8.39) | 47.32 (7.92) | 49.26 (8.37) | 48.75 (8.24) | 46.72 (9.81) | 48.77 (8.34) | 0.66 |
| **Male Gender** | 142 (53.6%) | 28 (65.1%) | 28 (66.7%) | 10 (47.6%) | 14 (56.0%) | 62 (46.3%) | 0.08 |
| **Race/Ethnicity** |  |  |  |  |  |  |  |
| Caucasian | 186 (72.4%) | 31 (73.8%) | 28 (68.3%) | 18 (85.7%) | 15 (62.5%) | 94 (72.9%) | 0.26 |
| African-American | 21 (8.2%) | 1 (2.4%) | 7 (17.1%) | 1 (4.8%) | 4 (16.7%) | 8 (6.2%) |  |
| Latino | 11 (4.3%) | 4 (9.5%) | 1 (2.4%) | 0 (0.0%) | 1 (4.2%) | 5 (3.9%) |  |
| Asian/API/Native American | 39 (15.2%) | 6 (14.3%) | 5 (12.2%) | 2 (9.5%) | 4 (16.7%) | 22 (17.1%) |  |
| **HCV Genotype†** |  |  |  |  |  |  |  |
| Genotype 1 | 175 (70.0%) | 17 (40.5%) | 15 (62.5%) | 16 (80.0%) | 35 (89.7%) | 92 (73.6%) | <0.0001 |
| Genotype 2 | 41 (16.4%) | 14 (33.3%) | 5 (20.8%) | 1 (5.0%) | 4 (10.3%) | 17 (13.6%) |  |
| Genotype 3 | 28 (11.2%) | 11 (26.2%) | 2 (8.3%) | 3 (15.0%) | 0 (0.0%) | 12 (9.6%) |  |
| Genotype 4 | 3 (1.2%) | 0 (0.0%) | 0 (0.0%) | 0 (0.0%) | 0 (0.0%) | 3 (2.4%) |  |
| Genotype 6 | 3 (1.2%) | 0 (0.0%) | 2 (8.3%) | 0 (0.0%) | 0 (0.0%) | 1 (0.8%) |  |
| **Baseline Fibrosis Stage** |  |  |  |  |  |  |  |
| 0 | 71 (26.8%) | 5 (11.6%) | 3 (12.0%) | 5 (23.8%) | 2 (4.8%) | 56 (41.8%) | <0.0001 |
| 1 | 68 (25.7%) | 11 (25.6%) | 7 (28.0%) | 2 (9.5%) | 11 (26.2%) | 37 (27.6%) |  |
| 2 | 70 (26.4%) | 18 (41.9%) | 3 (12.0%) | 9 (42.9%) | 10 (23.8%) | 30 (22.4%) |  |
| 3 | 30 (11.3%) | 5 (11.6%) | 9 (36.0%) | 3 (14.3%) | 8 (19.0%) | 5 (3.7%) |  |
| 4 | 26 (9.8%) | 4 (9.3%) | 3 (12.0%) | 2 (9.5%) | 11 (26.2%) | 6 (4.5%) |  |
| **Baseline Inflammation Grade‡** |  |  |  |  |  |  |  |
| 0 | 13 (5.2%) | 2 (4.8%) | 1 (4.2%) | 1 (5.3%) | 1 (2.9%) | 8 (6.2%) | <0.0001 |
| 1 | 75 (30.2%) | 5 (11.9%) | 7 (29.2%) | 6 (31.6%) | 1 (2.9%) | 56 (43.4%) |  |
| 2 | 131 (52.8%) | 30 (71.4%) | 12 (50.0%) | 8 (42.1%) | 21 (61.8%) | 60 (46.5%) |  |
| 3 | 28 (11.3%) | 5 (11.9%) | 4 (16.7%) | 4 (21.1%) | 10 (29.4%) | 5 (3.9%) |  |
| 4 | 1 (0.4%) | 0 (0.0%) | 0 (0.0%) | 0 (0.0%) | 1 (2.9%) | 0 (0.0%) |  |
| **BMI, Mean (SD)** | 27.92 (5.81) | 27.65 (5.77) | 29.62 (6.69) | 26.47 (4.95) | 28.61 (6.10) | 27.59 (5.57) | 0.35** |
| **Diabetes Mellitus** | 27 (10.2%) | 3 (7.0%) | 12 (28.6%) | 2 (9.5%) | 2 (8.0%) | 8 (6.0%) | 0.0009 |
| **Transfusion Before 1992** | 59 (24.5%) | 6 (16.7%) | 10 (27.0%) | 5 (27.8%) | 8 (36.4%) | 30 (23.4%) | 0.53 |
| **Number of Follow-up Images/Liver Biopsy, Mean (SD)** | 1.13 (1.24) | 0.84 (1.13) | 1.60 (1.25) | 1.29 (1.15) | 1.08 (1.15) | 1.06 (1.27) | 0.02** |
| **Cirrhosis during Follow-up** | 28 (11.7%) | 2 (5.1%) | 11 (35.5%) | 1 (5.3%) | 4 (18.2%) | 10 (7.8%) | 0.0008* |
| **HCC during Follow-up** | 14 (7.6%) | 1 (3.7%) | 6 (18.8%) | 0 (0.0%) | 0 (0.0%) | 7 (7.6%) | 0.11* |
| **Liver Transplant during Follow-up** | 12 (4.5%) | 1 (2.3%) | 6 (14.3%) | 1 (4.8%) | 2 (8.0%) | 2 (1.5%) | 0.009* |
| **Death during Follow-up** | 27 (10.2%) | 2 (4.7%) | 5 (11.9%) | 3 (14.3%) | 4 (16.0%) | 13 (9.7%) | 0.50* |
| **Years of Follow-Up, Mean (SD)** | 7.55 (4.09) | 7.24 (4.04) | 8.67 (4.38) | 8.74 (5.33) | 6.74 (4.08 | 7.27 (3.75 | 0.18** |
| **Treated Patients Only** | **N=131** |  |  |  |  |  |  |
| **Courses of Treatment** |  |  |  |  |  |  |  |
| 1 | 105 (80.2%) | 41 (95.3%) | 23 (54.8%) | 17 (81.0%) | 24 (96.0%) | NA | <0.0001* |
| ≥2 | 26 (19.8%) | 2 (4.7%) | 19 (45.2%) | 4 (19.0%) | 1 (4.0%) | NA |  |
| **Length of IFNα treatment (wk), Mean (SD)** | 44.82 (36.13) | 42.19 (17.40) | 62.05 (50.29) | 49.24 (24.47) | 14.26 (13.59) | NA | <0.0001** |
| **Therapeutic Regimen** |  |  |  |  |  |  |  |
| IFNα monotherapy | 14 (10.7%) | 0 (0.0%) | 10 (23.8%) | 3 (14.3%) | 1 (4.0%) | NA | 0.0008 |
| IFNα/RBV therapy | 117 (89.3%) | 43 (100.0%) | 32 (76.2%) | 18 (85.7%) | 24 (96.0%) | NA |  |

P-values were calculated from Chi-square test for category variables and ANOVA for continuous variables unless otherwise marked

* P-values were calculated from Fisher's Exact test

** Variable was rank transformed

[† 15 cases missing HCV genotype data](http://en.wikipedia.org/wiki/Dagger_(typography))

‡ 17 cases missing baseline inflammation grade
