# Supplementary material for: Nonresponse to Interferon-α Based Treatment for Chronic Hepatitis C Infection Is Associated with Increased Hazard of Cirrhosis
Source: PLoS One. 2013 Apr 25;8(4):e61568. doi: 10.1371/journal.pone.0061568 (PMC3636226; doi:10.1371/journal.pone.0061568)
Supplement: Table S2 — Risk Factors of Treated and Untreated Patient Groups (UCSF Cohort). (DOC) [file pone.0061568.s004.doc]

**Table S2. Risk Factors of Treated and Untreated Patient Groups (UCSF Cohort)**

| **Variable** | **Total** | **SVR** | **NR** | **Relapser** | **ETD** | **No Treatment** | **p-value** |
| --- | --- | --- | --- | --- | --- | --- | --- |
|  | **(N=265)** | **(N=43)** | **(N=42)** | **(N=21)** | **(N=25)** | **(N=134)** |  |
| **History of Heavy Drinking** | 89 (34.9%) | 13 (31.8%) | 6 (26.1%) | 10 (52.6%) | 17 (41.5%) | 43 (32.8%) | 0.21 |
| **Active IDU** | 1 (0.4%) | 0 (0.0%) | 0 (0.0%) | 0 (0.0%) | 0 (0.0%) | 1 (0.8%) | 0.51* |
| **Active Substance Use (non-IDU)** | 236 (89.1%) | 40 (93.0%) | 20 (80.0%) | 19 (90.5%) | 35 (83.3%) | 122 (91.0%) | 0.30* |
| **Current Methadone** | 8 (3.0%) | 0 (0.0%) | 2 (8.3%) | 1 (4.8%) | 1 (2.4%) | 4 (3.0%) | 0.30* |
| **History of Depression** | 47 (17.9%) | 6 (14.0%) | 2 (8.3%) | 3 (14.3%) | 10 (23.8%) | 26 (19.5%) | 0.62 |
| **PTSD** | 1 (0.8%) | 0 (0.0%) | 0 (0.0%) | 0 (0.0%) | 1 (2.4%) | 1 (0.8%) | 0.62* |
| **Social Stability** | 138 (80.2%) | 24 (80.0%) | 13 (76.5%) | 11 (68.8%) | 31 (91.2%) | 59 (78.7%) | 0.33* |
