# Supplementary material for: Nonresponse to Interferon-α Based Treatment for Chronic Hepatitis C Infection Is Associated with Increased Hazard of Cirrhosis
Source: PLoS One. 2013 Apr 25;8(4):e61568. doi: 10.1371/journal.pone.0061568 (PMC3636226; doi:10.1371/journal.pone.0061568)
Supplement: Table S3 — Predictors of Development of Cirrhosis Stratified by Fibrosis Stage (UCSF Cohort). (DOC) [file pone.0061568.s005.doc]

**Table S3. Predictors of Development of Cirrhosis Stratified by Fibrosis Stage (UCSF Cohort, N=239)***

| **Characteristics^‡^** | **Univariate Model** | **Multivariate Model^¶^** |
| --- | --- | --- |
|  | **HR^†^ (95% CI)** | **HR^†^ (95% CI)** |
| **Treatment outcome** | | |
| SVR | 0.83 (0.18 - 3.82) | 1.12 (0.12-10.33) |
| Nonresponder | 2.28 (0.93 - 5.59) | 5.90 (1.50-23.24) |
| Relapser | 0.31 (0.04 - 2.53) | 0.23 (0.02-2.27) |
| ETD | 2.94 (0.91 - 9.53) | 3.00 (0.70-12.84) |
| Never Treated | ref | ref |
| **Age at first liver clinic visit—per year increase^§^** | 1.02 (0.97-1.07) |  |
| **Male gender^§^** | 0.97 (0.45-2.07) |  |
| **Race^§^** | | |
| African American | 1.71 (0.56-5.21) |  |
| Latino | 0.86 (0.11-6.58) |  |
| Asian/API/Native American | 1.71 (0.66-4.46) |  |
| Caucasian |  |  |
| **HCV genotype 1 or 4^§^** | 0.79 (0.36-1.73) |  |
| **BMI—per unit increase** | 1.05 (1.00-1.11) |  |
| **Social Stability^§^** | 0.47 (0.19-1.21) | 0.23 (0.07-0.79) |
| **Bipolar or Thought Disorder^§^** | 2.43 (0.55-10.71) |  |

* Cox Proportional Hazards Models using time dependent covariate correcting for differences in waiting times from baseline to treatment initiation

^†^Hazard Ratio (HR) calculated using Cox Proportional Hazards Modeling

^‡^Interaction terms not shown

^§^Risk factors that significantly differentiate the treated from never treated groups

**^¶^**Final multivariate model after complete backwards stepwise selection
