# Supplementary material for: Nonresponse to Interferon-α Based Treatment for Chronic Hepatitis C Infection Is Associated with Increased Hazard of Cirrhosis
Source: PLoS One. 2013 Apr 25;8(4):e61568. doi: 10.1371/journal.pone.0061568 (PMC3636226; doi:10.1371/journal.pone.0061568)
Supplement: Table S4 — Predictors of Death or Liver Transplant Stratified by Fibrosis Stage (UCSF Cohort). (DOC) [file pone.0061568.s006.doc]

**Table S4. Predictors of Death or Liver Transplant Stratified by Fibrosis Stage (UCSF Cohort, N=265)***

| **Characteristics^‡^** | **Univariate Model^¶^** |
| --- | --- |
|  | **HR^†^ (95% CI)** |
| **Treatment Outcome** | |
| SVR | 0.24 (0.05-1.10) |
| Nonresponder | 0.43 (0.13-1.38) |
| Relapser | 0.80 (0.21-3.04) |
| ETD | 0.63 (0.18-2.20) |
| Never Treated | ref |
| **Age at baseline liver biopsy—per year increase** | 1.07 (1.02-1.12) |
| **Race^§^** | |
| African American | 0.43 (0.06-3.23) |
| Latino | 1.38 (0.18-10.48) |
| Asian/API/Native American | 1.14 (0.44-2.96) |
| Caucasian | ref |
| **Substance Use** | |
| History of Heavy Drinking^§^ | 1.58 (0.69-3.62) |
| Current Methadone | 3.90 (0.87-17.42) |
| **BMI—per unit increase** | 0.93 (0.87-1.00) |
| **Social Stability^§^** | 0.58 (0.21-1.56) |
| **Length of all IFNα treatment—per week increase** | 0.99 (0.97-1.00) |

* Cox Proportional Hazards Models

^†^Hazard Ratio (HR) calculated using Cox Proportional Hazards Modeling

^‡^Interaction terms not shown

^§^Risk factors that significantly differentiate the treated from never treated groups

**^¶^**There were no significant covariates in multivariable Cox Proportional Hazards Models
