# Supplementary material for: Nonresponse to Interferon-α Based Treatment for Chronic Hepatitis C Infection Is Associated with Increased Hazard of Cirrhosis
Source: PLoS One. 2013 Apr 25;8(4):e61568. doi: 10.1371/journal.pone.0061568 (PMC3636226; doi:10.1371/journal.pone.0061568)
Supplement: Statistical Methods S1 — Details of Construction and Reduction of Statistical Models. (DOC) [file pone.0061568.s007.doc]

**Supporting Information S1**

To build the adjusted (multivariate) hazard models, we first examined the univariate statistics, looking at the contribution of each identified risk factor to the probability of developing cirrhosis or of dying within the time-censored follow-up period. We chose predictors that had a *p*-value of 0.2 for inclusion in initial models. We initially used a deliberately large *p*-value to examine the net effect of all plausible predictors. We then subjected these models to two types of assessment. We examined the *p*-values of all predictors in the resultant models and eliminated, one by one, those with the highest *p*-values. At each elimination step we checked for confounding by looking at the parameter estimate for the major predictors—in this case treatment response (SVR, relapse, and NR)—both before and after the examined variable was eliminated from the model. If the treatment response parameter estimate changed by 10% or more, we determined that the variable was a significant confounder and it was left it in the model. We then repeated this process working our way down from the predictor with the next highest *p*-value until all remaining predictor variables had a *p*-value ≤ 0.05. Selected known risk factors were forced into the model despite having *p*-values greater than 0.2. After completing this process, we performed stepwise elimination in SAS, using Proc PHREG, first entering the full model, to confirm our results.

We used two strategies to adjust for the non-random distribution of characteristics differentiating treated from untreated patients, including age at initial liver biopsy, race/ethnicity, HCV genotype, active alcohol abuse, other substance use, psychiatric comorbidities, adequacy of social support, and liver clinic non-compliance. These factors were reassessed individually in univariate models in the time-to-event analyses and incorporated in the full multivariate models, as appropriate, where they were tested for confounding in stepwise elimination (described above) and stratified by fibrosis score at initial biopsy. As an alternative strategy, propensity scores were derived from composite risk factor profiles and substituted into the final multivariate survival models to estimate an average effect from the factors related to treatment selection using previously described methods [1,2,3]. Adjusted hazard ratios resulting from the two approaches were compared.

1. Etzioni R, Pepe M, Longton G, Hu C, Goodman G (1999) Incorporating the time dimension in receiver operating characteristic curves: a case study of prostate cancer. Med Decis Making 19: 242-251.

2. Luo Z, Gardiner JC, Bradley CJ (2010) Applying propensity score methods in medical research: pitfalls and prospects. Med Care Res Rev 67: 528-554.

3. Shah BR, Laupacis A, Hux JE, Austin PC (2005) Propensity score methods gave similar results to traditional regression modeling in observational studies: a systematic review. J Clin Epidemiol 58: 550-559.
