# Supplementary material for: Nonresponse to Interferon-α Based Treatment for Chronic Hepatitis C Infection Is Associated with Increased Hazard of Cirrhosis
Source: PLoS One. 2013 Apr 25;8(4):e61568. doi: 10.1371/journal.pone.0061568 (PMC3636226; doi:10.1371/journal.pone.0061568)
Supplement: Replication Cohort S1 — UCSF Medical Center Liver Clinic. (DOC) [file pone.0061568.s008.doc]

**Supporting Information S2**

We performed a retrospective medical records review of consecutive, chronic HCV patients presenting for treatment consideration at the University of California, San Francisco (UCSF) gastroenterology faculty practice from January 1, 1991 to June 30, 2008. A waiver of patient consent was obtained from the UCSF Institutional Review Board. The inclusion criteria were identical to those used with the SFVA cohort. At the time of treatment consideration visit, subjects had to satisfy the following criteria: age > 18 years, a diagnosis of chronic HCV viremia, availability of at least one pretreatment liver biopsy report, and follow-up by an affiliated physician at least one year after the baseline biopsy. Exclusion criteria included: HIV-1 and HBV co-infection, decompensated cirrhosis or prior liver transplant, or unavailable information on prior treatments (including duration, course, and outcome). The methods used for data validation, cleaning and analysis were identical to those used with the SFVA cohort.

Results: The demographic and clinical characteristics of the UCSF cohort are presented in Table S1. The mean age of the UCSF patients was 48 years. Forty-seven percent of the patients in this cohort were female. Seventy-two percent of UCSF patients were Caucasian, 8.2% African American, 4.3% Latino and 15.2% were Asian/Pacific Islander or Native American. In univariate Cox proportional hazards models for time- to-cirrhosis, treatment nonresponders had a marginally significantly increased hazard of cirrhosis (HR = 2.28, CI 0.93-5.59) compared to never treated patients. Other risk factors significant for the hazard of cirrhosis included incremental increases in age and BMI (HR = 1.02, CI 0.97-1.07 and HR = 1.05, CI 1.00-1.11, respectively). In multivariate hazards models, where factors that significantly differentiated the treated from never treated groups were forced into the models, treatment nonresponse became a significant predictor of cirrhosis (HR = 5.90, CI1.50-23.24), while adequate social support emerged as significantly protective (HR = 0.23, CI 0.07-0.79) (Table S3). In survival analysis, SVR achieved a marginally protective effect (HR = 0.24, CI 0.05-1.10), while age had an incremental statistically significant hazard (HR = 1.07, CI 1.02-1.12). In stratified multivariate analysis adjusting for psychosocial, clinical, and behavioral risks, there were no significant predictors of survival (Table S4).
